# On the Physiological and Psychological Phenomena of Dreams and Apparitions

**Published:** 1856-07-01

**Authors:** 


### ART. IV.—ON THE PHYSIOLOGICAL AND PSYCHOLOGICAL PHENOMENA OF DREAMS AND APPARITIONS.

[No. I. of a Series.]

MAN is regarded as a living microcosm. The organic machinery which regulates his bodily and mental functions are of two kinds:—

Firstly, those which induce the involuntary functions of animal life, as the heart and other organs associated with the circulation of the blood, and the lungs, by which its vital quality is preserved; besides which there are the various organs of secretion and excretion, by which the body and its different parts are kept in repair, or which are essential to remove poisonous matter from the vital fluid itself, which supplies the material for this renovating process.

Secondly; the voluntary functions, which are essential to conserve to the wants of the individual—animal, moral, and intellectual, or which conduce to his relaxation, refinement, and elevation, as the highest in the scale of created beings, and which conjointly constitute him an organic entity with social, domestic, responsible, percipient, and reflective powers, and through which he preserves his relations to both the outer and the inner world.

It is the voluntary powers, or those which are appreciated by consciousness, that must occupy a few brief remarks, as essential to comprehend the views to be submitted in this Essay.

Before we can explain the phenomena of dreams, it is important to understand what is the final cause of sleep. We may premise, that the machinery of our complicated body is worked by a certain *stimulus*, which we call, for practical purposes, nervous fluid, or *vis nervosa*, and which, from evidence to be submitted, seems to depend on the brain for its elaboration.

This fluid, in a healthy person, is supplied to all the organs essential to digestion, assimilation, locomotion, and so forth. And when the body and mind are in a normal condition, the distribution of this vital principle will be in the due proportion to the requisite wants of every organ essential for the functions indicated.

Yet it is a matter of experience, that the nervous fluid may be exhausted by too great mental occupation, in which case the digestion is enfeebled, and the organic functions seriously deranged; or if this important stimulus is appropriated in too great proportion by the muscular system, as in excessive exercise; that in either case exhaustion is induced, and nature has rendered the sense of fatigue, consequent on such conditions, to be followed by a desire for repose.

Let us trace the obvious and salutary results. During the absolute rest of the organs of volition, and the nervous centres by which all actions are performed, there is what is called profound sleep. The brain ceases for a time to be the instrument of thought or emotion, although, as a great galvanic battery, it seems to be elaborating a fresh supply of the vital power (nervous fluid), and, by means of its continuous conductors—the nerves—distributing it to every organ of the body. And thus, when the sleeper awakes, he is refreshed and invigorated.

But if the sleep is imperfect and partial, these results do not follow, and there is experienced a sense of lassitude and discomfort. It is in such unsound sleep that dreams occur.

That there is nothing speculative in these opinions as to the actual condition of the brain, in sound and in partial sleep, we cite a case quoted by Dr. M'Nish in his "Philosophy of Sleep," as furnishing conclusive evidence on these interesting phenomena:—

"Dr. Perquin, a French physician, records the fact now quoted. It fell under his notice in one of the hospitals of Montpelier, in the year 1821. A female of the age of twenty-six had lost part of her scalp, skull-bone, and dura-mater, under a malignant disease (syphilis), which had been neglected. In consequence of these injuries, a portion of the brain had been exposed, and admitted of being inspected.

"When she was in a dreamless sleep, her brain was motionless, and lay within the cranium. When her sleep was imperfect, and she was agitated by dreams, her brain moved and protruded within the cranium, forming a cerebral hernia. In vivid dreams, reported as such by herself, the protrusion was considerable. And when she was perfectly awake, especially if engaged in active thought or sprightly conversation, it was still greater. Nor did the protrusion occur in jerks, alternating with recessions, as if caused by the impulse of arterial blood. It remained steady while conversation lasted."*

This fact, and other similar ones, prove that during profound sleep the active condition of the mental functions ceases; although it is most probable that the base of the brain may continue to supply nervous energy to the heart and lungs. But during the dreaming state, some of the mental powers indicate their activity by motions in the cerebral organs on which they depend for their manifestations.

This knowledge furnishes some data for our subsequent opinions on the philosophy of dreams and ghosts, and which, if rejected, leave these subjects more curious than of any practical advantage to comprehend a true philosophy of mind; for, without such information, there is a liability to form the most discrepant theories, as may be verified by examining the attempted explanations of these phenomena by many previous writers.

Thus Addison regards "dreams as the soul's relaxation after she is dismembered of her machinery, and that she is then the theatre, the actor, and the beholder."

And Dryden furnishes not any better solution when he says,—

"Dreams are the interludes which fancy makes;
When Monarch Reason sleeps, this mimic wakes;
Compounds a medley of disjointed things—
A mob of cobblers, and a court of kings.
Light fumes are merry, grosser fumes are sad,
Both are the reasonable soul run mad;
And many monstrous forms in sleep we see,
That never were, nor are, nor e'er can be."

Dugald Stewart's theory of dreaming is, that in sleep we have lost, or nearly so, all volition over the bodily organs; but that those mental powers which are directly important for our volition may retain a certain degree of activity.

* We may incidentally remark, that if there were but this one solitary instance on record, its importance in connexion with the subject under consideration is as valuable to the student of the philosophy of dreams, as the accident narrated by Dr. Beaumont, of America, (cited by Dr. Andrew Combe, in his work on Digestion,) has proved in reference to gastric phenomena. In both instances there is furnished important demonstrations of the respective functions of the brain and stomach; and the means of confirming or correcting the speculations of physiologists who had previously written on both subjects.

The writer of the Article *Dreams* in the "Encyclopædia Britannica," asks—

"What parts of the human being are active, and what dormant, when he dreams? Why does not he always dream when asleep? Or why dreams he at all? Do any circumstances in our constitution, situation, and peculiar character determine the nature of our dreams?"*

Abercrombie speaks of four varieties of the dream: 1st. Wrong associations of new events. 2nd. Trains of thought from bodily associations. 3rd. Revival of associations. 4th. Casual fulfilment of a dream. We do not essentially differ from these divisions, but do not recognise the last. Nor does this author give any lucid elucidation of the predisposing causes for these various phases of dreaming. And if we look for more definite notions among ancient writers, including Plato, we shall be greatly disappointed. Certainly the latter has treated on these phenomena, and speaks of many kinds of dreaming, from the imagination half asleep, to those more painful kinds which he terms ephialtes (night-mare). And an anonymous writer says of dreams:—

"These nocturnal phantasmata, which disturb the soft embraces of Morpheus with their playful and visionary forms, are suggested often by pain, by sounds, and various bodily sensations, in the same manner as are trains of waking thoughts."

The apparent solution to what seems otherwise a mass of ambiguities, can only be found in a number of well-observed cases, based on the clear and lucid difference of physiological and psychological phenomena; and this result can only be ensured when we are guided by a sound system of mental philosophy in harmony with man's organization. And the first essential for the absolute comprehension of our subject is to admit the rationality of Gall's theory of the brain,—that it is not a single organ, but that its different portions are the instruments by which are manifested feelings, emotions, perceptions, and so forth.

If, on the contrary, we retain the earlier views of physiologists, and deem the whole brain essential for every sentiment or intellectual operation, we have not any definite data to aid us in our investigation, or to comprehend the various *phases* of temper, and disposition, and mental capacity which exist in different individuals, and mark them so perfectly during the waking state, and which preserve many of their specialities in dreams. And, without an admission of the compound nature of the mental

* We shall, in the course of this essay, answer these questions; or, in other words, our solution to these mental riddles, will, in a measure, reply to these queries.

machinery, little could be practically done in the treatment of many forms of insanity, which are proved to result from abnormal states of the brain.

It is, therefore, important for our purpose to offer certain presumptive evidence in favour of Gall's theory, as furnishing preliminary data for analysing the phenomena of dreams, and the examples which will be subsequently submitted as illustrations.

We need not cite the mass of indubitable evidence of "stubborn facts," which have led to the inductive inference that the brain *is* a compound organ,—that its various functions act purely in obedience to physiological laws, and yet there is nothing in these views which are opposed to our intuitive notions of a soul. All that is affirmed, then, by these views is simply that so long as there exists a connexion between the body and the soul in this life, the attributes of the latter must be manifested through the organization. Or, in other words, that the soul uses the brain for mental purposes, just in the same way that it uses the eyes to see with, and the muscles for volition. And finally, that its *unity* is not implicated because we speak of various feelings, sentiments, perceptions, and so forth, any more than is that of the body because its operations are compound, and the results diversified.

There is, however, additional evidence that the brain and its parts perform different and distinct functions, from its anatomical structure and great complexity in man. This complexity may be demonstrated by a comparison with the brains of other animals, which are less so, in different degrees, in a descending scale; or if we commence with the simplest form of the nervous system, and ascend in a regular series. Thus, the worm has not a nervous centre (a brain), and we perceive that its functions are purely automatic. And as we rise in the scale of being, even when but few instincts are manifested with volition, there is found a simple brain, and the cerebral mass increases in complexity more and more, in the ratio of the number of the mental functions each class of beings have to perform, until in man this complexity is the greatest, and his superiority is commensurate.

The mental faculties of man are divided into four genera—1st. The animal propensities essential to man's terrestrial wants. 2nd. The moral attributes, which are of an elevating and refining tendency, and control any overt acts of selfishness. 3rd. The external senses. 4th. The intellectual faculties.

The *senses* are regarded as the *media* between the world without, and inner or spiritual world within, whilst the mind alone gives us consciousness of our actual existence.

There is also an anatomical fact in reference to the brain, which is too important to be unheeded, as it is essential to comprehend some of the mental phenomena we shall have to refer to in explaining certain peculiarities in the dreaming state, and in ghost-seeing—that the brain is double, and like the nerves of sense, possesses an important purpose. The final cause may be, that if one set of organs are fatigued, the other may exercise their respective functions. And in injuries from accident or disease in one hemisphere, the mental operations may be carried on by the healthy one ; in the same manner as when an eye is destroyed by some casualty, the other may continue the function of vision.

With these preliminary considerations, we may proceed to discuss the views to be submitted in this essay. And, assuming that we have made it evident, that when the external senses and the brain are in a state of that comparative negation which we designate by a condition of perfect rest, sleep the most profound is the certain consequence. But if only parts of the brain rest, and the external senses are easily excited by their respective *stimuli*, then dreams are almost a certain result. And their coherence or incoherence will depend on the number, more or less, of those faculties which are, in the waking condition, essential for obtaining clear perceptions, independently of any accidental association.

> "Lulled in the countless chambers of the brain,
> Our thoughts are linked, by many a hidden chain,
> Awake but one, and lo! what myriads rise,
> Each stamps its image, as the other flies."

That these views are in accordance with accurate observation, we may confirm by noticing the effect of an over vigilance of the mental faculties, that in such cases sleep is banished, and the converse phenomena take place of those which occur from a normal result of certain healthy exercises. The vigilant condition is thus graphically described in Henry the Fourth's beautiful soliloquy on *Sleep*.

> "O gentle sleep!
> Nature's soft nurse, how have I frighted thee,
> That thou no more will weigh my eyelids down,
> And steep my senses in forgetfulness."

The predisposing causes of dreams may be various, but they are all invariably referrible to some conditions of the body—its position, irregular or excessive circulation; or to some conditions of the brain, or the external nerves.*

* Although we refer dreams to cerebral phenomena, yet we think with an anonymous writer that this doctrine does not invalidate the proof of the prophetic use God may have formerly made of them ; for Omnipotence may excite material organs in a definite manner, so as to convey true prophecies. The phantoms them-

Wolfius and Forney supposed that dreams never arise in the mind, except in consequence of some of the organs of sensation having been previously excited. But these views are only part of the truth, unless by a latitude of speech, the cerebral organs are included.

We shall consider the subject of dreaming under four heads—

1st. When the primary impression is made on the sense of sight, smell, taste, touch, and hearing, as the first division of what we may term suggestive causes.

2nd. Physical sensations, as cold, heat, currents of air, et cetera, as inducing a second form of suggestive causes.

3rd. When the mental faculties are some of them in a state of activity, from some abnormal condition of health.

4th. When a similar, or aggravated condition exists, from narcotics, or stimulants, with an active temperament.

And, lastly, we shall indicate some curious conditions of the muscles, either as inducing dreams, or as resulting from some circumstance connected with them.

We have collected a number of interesting instances which clearly prove that when the mind is in the transition state between sleeping and wakefulness, that during this period of imperfect consciousness, if any strong impression is made on the organs of the external senses, such impression is a suggestive source of some form of dreaming. But then there must be some susceptibility to be easily influenced by the respective stimuli, as in profound sleep the "senses," like the brain, are steeped in forgetfulness. A few examples will better explain our meaning than any mere theoretic views.

Blind Man of Barnsley.—Mr. ——, a retired tradesman, at whose house the writer resided for a brief period, was totally blind, except being sensible of the difference of a light or a dark apartment. He had lost his sight for some years, but it was after his children had, most of them, grown up to be young men and women. During the previous portion of his life, he had been in the habit of taking active exercise—hunting two or three times a week. When at his house at the time now mentioned, he was doomed to remain within doors, or sit, during fine weather, in the garden. The circumstance to be related occurred on a beautiful morning in October. He had had a chair placed for him with his face in a southern aspect, the bright sun shining on him. And there he sat in deep thought, smoking his pipe, musing o'er his past existence. He, however, soon fell selves may be referrible to motions of the organs of the brain, like ocular spectra in the retina, or the imaginary sounds and noises that some nervous people hear. But the coincidences between the dream and the event which it seemed to predict, constitute the astonishing part, and render them miraculous.

into a sound sleep, just as we entered the garden. He continued so for some time, and moved himself, whilst his expression then became more pleasing than usual, and it was manifest that he was dreaming, and we watched with great interest the varying changes of his well-formed features. Instead of his ordinary appearance of discontent, or a merely blankless expression, he appeared to be agreeably excited. At length we observed tears flowed from his sightless eyes, and trickled down his face, and he soon awoke to all the reality of his situation. He sighed like one who experienced great mental agony. "You have been dreaming," we said. "Yes, sir, and I felt more happy than I have done for years, for I thought my sight was restored; and that I once more beheld the faces of my dear wife and family." He paused, and then continued, "Would that this sleep had continued, for now the contrast of my painful condition makes me most miserable!" And the poor old gentleman cried in a most piteous manner.

It is evident that the bright rays, with their genial warmth, had suggested the dream we have narrated. If, however, he had been altogether incapable of being affected by the stimulus of light, this could not have occurred; and whilst it confirms a physiological law, it induces certain reflections on psychological phenomena, inasmuch as he had a perfect consciousness of a condition of mind he could not realise from any memoretic effort.

On the SENSE OF SMELL, as a suggestive cause, we cite the following, from many in our note-book. It was furnished by the dreamer. "On one occasion, during my residence at Birmingham, I had to attend many patients at Coventry, and for their accommodation I visited that place one day in every week. My temporary residence was at a druggist's shop in the market-place. Having on one occasion, now to be mentioned, a more than usual number of engagements, I was obliged to remain over night, and a bed was procured for me at the residence of a cheesemonger in the same locality. This house was very old, the rooms very low, and the street very narrow. It was summer time, and during the day the cheesemonger had unpacked a box or barrel of strong, old American cheese. The very street was impregnated with their odour. At night, jaded with my professional labours, I went to my dormitory, which seemed filled with a strong cheesy atmosphere, which affected my stomach greatly, and quite disturbed the biliary secretions. I tried to produce a more agreeable atmosphere to my olfactory sense by smoking cigars, but did not succeed. At length, worn out by fatigue, I tried to sleep, and should have succeeded, but for a time another source of annoyance prevented my doing so—for in the old wall behind my head, against which my ancient bedstead stood, there were numerous rats gnawing away in real earnest. The crunching they made was, indeed, terrific; and I resisted the drowsy god from a dread that these voracious animals would make a forcible entrance, and might take personal liberties with my flesh!

"But at length 'tired nature' ultimately so overpowered me, that I slept in a sort of fever. I was still breathing the cheesy atmosphere, and this, associated with the marauding rats, had so powerfully affected my imagination, that a most horrid dream was the consequence. I fancied myself in some barbarous country, where, being charged with a political offence, I was doomed to be incarcerated in a large cheese. And although this curious prison-house seemed most oppressive, it formed but part of my sufferings. For scarcely had I become reconciled to my miserable fate, than, to my horror, an army of rats attacked the monster cheese, and soon they seemed to have effected an entrance, and began to fix themselves in numbers on my naked body. The agony I endured was increased by the seeming impossibility to drive them away, and fortunately for my sanity, I awoke; but with a hot head and throbbing temples, and a sense of nausea from the increasingly strong odour of the cheese."

It is worthy of a passing reflection, that although the whole dream adventure was altogether improbable, yet it must be remembered that all the auxiliaries were present to the waking thoughts of the dreamer, and hence the exaggerated and painful associations were induced, in all probability from the *odour* and *gnawing* being still appreciated in some degree in the feverish and partial sleep.

That the SENSE OF HEARING may be also suggestive of a dream we have many examples, but shall merely quote one instance. The celebrated sculptor, Mr. P—— H—— once related to us an amusing instance: "His brother dreamt, just before rising in the morning, that he had to see a Mr. Jones, and that, in order to do so, he walked up a flight of steps leading to a very large house, intending to inquire for him, but as soon as he asked the servant if his friend resided there, a little pert, dwarf-like figure suddenly started up on one side of him, and asked, in a shrill, squeaking voice, 'What Jones? What Jones?' Vexed at this impertinent intrusion, the dreamer determined to be more cautious, and going up to the next house, he repeated his question in a lower tone, when the same rude, ugly dwarf, repeated his questions, 'What Jones? What Jones?' And instead of noticing this officious fellow, he turned away in anger, and awoke! when he heard some one passing under his window, calling out 'Hot rolls! hot rolls!' And his partially awaked faculties having caught these sounds, he had in a brief period produced the incidents of the dream just related."

We could narrate many curious dreams induced by some unpleasant effect on the SENSE OF TOUCH, and as an example, by way of illustration, select the following. A gentleman, of a nervo-bilious temperament, was much out of health from too much mental employment, and was ordered by his physician to take a pill, and in attempting to swallow it he did not succeed, but lodged it on the rim of the pharynx, and caused him great annoyance. He tried to dislodge it by putting his fingers down his throat, but he could not succeed. So, after repeatedly coughing, until he was tired out, he fell asleep. Soon after he had done so he had a most painful dream. We shall relate it in his own words: "It appeared to me that I was in some large town on the Continent, and a perfect stranger to every one; that I had strolled out from the inn, although the night was dark and the streets ill-lighted; and when I attempted to retrace my steps and return, the farther the inn seemed from me. At length, hearing footsteps, I followed, hoping to gain some information or assistance. And, to my horror, I found myself in a narrow court, without any thoroughfare. After calling to the invisible being, he seemed to stop, and before I could ask a question, he seized me by the throat, and produced a sensation of pain. I attempted to speak, when the inhuman wretch thrust his hand in my mouth, and nearly choked me; but in the agony of my situation I made a desperate effort to bite this fleshy plug, and made the cowardly wretch scream out as he felt the dental incision, and this scream awoke me. The pill remaining in its position, the irritation of which and my own previous experiment to dislodge it, had induced the painful train of thought of my short and feverish dream, which could not have lasted more than a few minutes."

That the SENSE OF TASTE is suggestive of dreams might be proved by many examples. But we may merely remark that this, like those already examined, acts as a predisposing cause, or as suggestive of dreaming. For instance, if a person goes to bed hungry, there is a craving for food, and the probability is that he will dream of feasting. And most likely the repast will consist just of such viands or drinks as are most agreeable to the palate of the individual during his perfect consciousness. And by a reflex action of the cerebral organs actively exercised, the sense of taste will be affected with similar perceptions of flavours as are in reality experienced during the process of mastication and deglutition. A friend of ours, who was particularly fond of soups, went to bed hungry, and dreamt he was eating some rich preparation of meat, the smell of which induced him to eat it rather quickly; when he dreamt that some of it went "the wrong way," as it is called, and almost choked him; and he awoke, actually coughing. The latter spasmodic effort, with his feeling a want of food, induced the kind of dream; but which confirm the previous observations incidentally made, that he still fancied, though only for a brief time, that he smelt the savoury stew or soup, which in his dream he had so much enjoyed.

We cannot conclude this section without observing that none of the latter forms of dreaming must be confounded with ephialtes or *incubus*, as we shall, when speaking of the latter, prove that there are so many phenomena connected with the *incubus*, as clearly distinguish it from those trains of thought induced by those partially disturbing influences which result from some of the external senses being affected by their respective *stimuli*.

In order to preserve some connexion in the subject under consideration, we shall offer a few brief observations on the second section—viz., that the physical sensations of cold and heat are suggestive of dreaming; and although we have more interesting phenomena to explain in the course of this essay, yet as external agents which affect the circulation of the blood, we could not reject them, and hence this is the fitting place to fairly discuss them.

It is obvious, on the slightest reflection, that if *cold* or *heat* sensibly affect the body, their effects must be cognized by the mental faculties. For in many abnormal states of the brain, insane persons have had portions of the feet burnt off without any apparent consciousness of pain.* But if the mind retains its ordinary functions, whatever affects the body will suggest trains of thought, even in partial sleep, and which dreams will be fashioned and connected with the physical agent which was the suggestive cause. We must premise that neither the heat or cold must be experienced in any extreme degree, or else it may produce a sedative effect on the brain, and there may be great danger, without any consciousness of it.

There are many cases on record of heat and cold inducing dreams, mentioned by writers as early as Macrobius; and some of our modern physiologists have made observations of a similar kind. A literary man, who had a most sensitive organization, related a rather amusing instance of the effects of cold, in suggesting a most annoying adventure to one of his thin-skinned tendency. He thus describes it: "The other morning my wife rose very early, and being unwell, I decided to take another turn, and soon fell asleep, but so imperfectly, that a succession of dreams haunted my imagination. One of them I submit to you

* One instance is so remarkable that we cannot resist mentioning the circumstance. It occurred in Bedlam, some years since. A man laid his feet on the fire, and kept them there until they were perfectly carbonized, without apparently suffering any inconvenience during the ignition.

as an interpreter of these phantasmata. Methought that the butcher had sent a joint different from the one we required, and that I determined to go back with it myself, when, to my horror, a number of persons followed me, and seemed discussing the point whether or not I was demented. 'Surely,' said one, 'he must be so, to go out without stockings this bitterly cold, frosty morning!' 'Has he been drinking?' said another; 'for a more robust man than he is could not be guilty of such an act with impunity.' Great was the sense of shame which oppressed me, and I hurried on, not daring to face my censors, and thought to escape them, when I heard some little girls and great girls titter, and then laugh most heartily as they exclaimed, 'What indelicacy, et cetera.' This second adventure pained me so much, that I awoke, and found the bed-clothes off my legs, which were almost painfully cold, having a tingling sensation from suppressed circulation." In this instance, and others we could cite, the predisposing cause was the cold, worked up into an adventure by the half-waking mental faculties.

A gentleman whom we knew told us that he had had a long walk, and had eaten a hearty supper. The night was cold, in consequence of which he had a number of blankets put on the bed; and which rendered him heated and feverish. These physical conditions gave rise to the following curious dream. He said that he fancied that he was walking on a summer evening, and felt a sense of suffocation, as there was not a breath of air; that the clouds had gathered in dense masses, and that whilst suffering from the oppressive heat, some large brown "cockroaches" flew against him with such force, as to induce severe pain wherever they touched. To avoid them he hurried to bed, but even there these tormentors pursued him, and at length they swarmed in such quantities, as literally to cover the pillows. In a state of terror and disgust he awoke. He was lying on his side, in an unnatural heated condition. This, he remarks, suggested the phenomena in reference to the weather, in which all unities were kept up, but highly exaggerated. The "cockroaches," however, puzzled him, until he recollected that, prior to his going to bed, one of his daughters read out a portion of the Memoirs of Benvenuto Cellini, in which he had described his journey from France, that he and his party were overtaken in a storm, in which the hail-stones were the size of lemons! His mind had retained the marvellous account, and in his dream he had changed the gigantic hail-stones into the unnatural-sized "cockroaches," as more in harmony with his sensations.

We may allude to physical agencies again, in the section on vivid reminiscences in dreams.

(*To be continued.*)

